# Modern methods in peach (Prunus persica) genome research

**DOI:** 10.18699/vjgb-25-39

**Published:** 2025-06

**Authors:** I.V. Rozanova, E.A. Vodiasova

**Affiliations:** Federal Research Center the N.I. Vavilov All-Russian Institute of Plant Genetic Resources (VIR), St. Petersburg, Russia The Nikitsky Botanical Gardens – National Scientific Centre of RAS, Nikita, Yalta, Republic of Crimea, Russia; The Nikitsky Botanical Gardens – National Scientific Centre of RAS, Nikita, Yalta, Republic of Crimea, Russia

**Keywords:** Prunus persica, GWAS, selection, genotyping, SNP, Prunus persica, GWAS, селекция, генотипирование, SNP

## Abstract

Peach (Prunus persica (L.) Batsch) is one of the main agricultural stone fruit crops of the family Rosaceae. Modern breeding is aimed at improving the quality of the fruit, extending the period of its production, increasing its resistance to unfavorable environmental conditions and reducing the total cost of production of cultivated varieties. However, peach breeding is an extremely long process: it takes 10–15 years from hybridization of the parental forms to obtaining fruit-bearing trees. Research into peach varieties as donors of desirable traits began in the 1980s. The first version of the peach genome was presented in 2013, and its appearance contributed to the identification and localization of loci, followed by the identification of candidate genes that control the desired trait. The development of NGS has accelerated the development of methods based on the use of diagnostic DNA markers. Approaches that allow accelerating classical breeding processes include marker-oriented selection (MOS) and genomic selection. In order to develop DNA markers associated with the traits under investigation, it is necessary to carry out preliminary mapping of loci controlling economically desirable traits and to develop linkage maps. SNP-chip approaches and genotyping by sequencing (GBS) methods are being developed. In recent years, genome-wide association analysis (GWAS) has been actively used to identify genomic loci associated with economically important traits, which requires screening of large samples of varieties for hundreds and thousands of SNPs. Study on the pangenome has shown the need to analyze a larger number of samples, since there is still not enough data to identify polymorphic regions of the genome. The aim of this review was to systematize and summarize the major advances in peach genomic research over the last 40 years: linkage and physical map construction, development of different molecular markers, full genome sequencing for peach, and existing methods for genome-wide association studies with high-density SNP markers. This review provides a theoretical basis for future GWAS analysis in order to identify high-performance markers of economically valuable traits for peach and to develop genomic selection of this crop.

## Abstract (ru)

Персик (Prunus persica (L.) Batsch) – одна из основных сельскохозяйственных плодовых косточковых культур семейства розоцветных. Современная селекция направлена на улучшение качества плодов, расширение сроков их получения, создание сортов с устойчивостью к неблагоприятным условиям среды и снижение общих затрат на производство культивируемых сортов. При этом селекция персика – долгий процесс: от гибридизации родительских форм до получения плодоносящих деревьев проходит 10–15 лет. Исследования сортов персика как доноров желаемых признаков начались с 1980-х годов. Первый вариант генома персика был представлен в 2013 г., и его появление способствовало определению и локализации локусов, с последующим обнаружением генов-кандидатов, под чьим контролем находится хозяйственно ценный признак. Развитие методов NGS ускорило продвижение подходов, основанных на применении диагностических ДНК-маркеров. К таким подходам, позволяющим ускорять процессы классической селекции, относятся маркер-ориентированная селекция и геномная селекция. Для того чтобы разработать ДНК-маркеры, ассоциированные с изучаемыми свойствами, необходимо провести предварительное картирование локусов, контролирующих хозяйственно ценные признаки, создать карты сцепления. Работы по пангеному показали необходимость анализировать большее количество образцов, так как до сих пор не хватает данных для нахождения полиморфных областей генома. Развиваются подходы использования SNP-чипов и методов генотипирования через секвенирование (GBS, genotyping-by-sequencing). В последние годы для обнаружения локусов генома, ассоциированных с хозяйственно ценными признаками, активно применяется метод полногеномного анализа ассоциаций (GWAS, genome-wide association studies), для использования которого требуется скрининг больших выборок сортов по сотням и тысячам SNP. Цель настоящего обзора – систематизация и обобщение основных достижений в области геномных исследований персика за последние 40 лет: построение карт сцепления и физических карт, получение различных молекулярных маркеров, полногеномное секвенирование для персика, а также описание существующих работ полногеномных исследований ассоциаций с маркерами SNP высокой плотности. Этот обзор обеспечивает теоретическую основу для проведения GWAS с целью выявления высокоэффективных маркеров хозяйственно ценных признаков для персика и развития геномной селекции этой культуры.

## Introduction

Peach (Prunus persica L.) is one of the main agricultural stone crops of the temperate zone, consumed fresh and processed, contains high amounts of vitamins, minerals, fiber and antioxidant compounds, while being low in calories and therefore excellent for dietary menus. As a species, it originated about 2.5 million years ago in the southwestern Tibetan Plateau region of China, from where its domestication began 4,000–5,000 years ago (Yu Y. et al., 2018) (see the Figure).

**Fig. 1.**
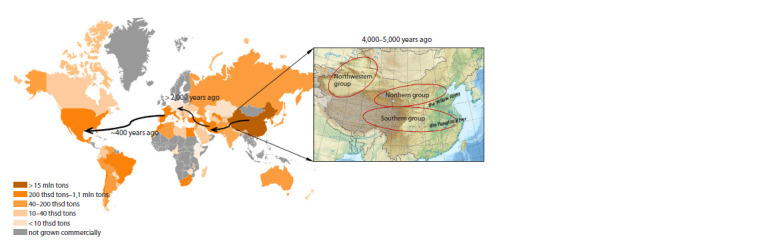
Distribution and origin of P. persica. The arrows show the distribution routes of the peach. Countries where the peach currently grows and its production level are shown according to the color scheme. Origin of peach in China: the Southern group spans the Yangtze River; the Northern group is along the Yellow River; the third group originated in northwest China.

The current peach gene pool is divided into three groups that are characterized by differing climatic growing conditions (see the Figure). These groups originated in China in different geographical regions. The Southern group originated in climates with mild winters and hot, humid summers. These peaches have a flat shape and are characterized by a slightly acidic “honey” flavor. The Northern group included peach genotypes found in regions with cold winters and hot, dry summers. These peaches are generally resistant to drought and cold, but are not adapted for growth in southern areas. The third group is found in the arid northwest of China. It includes nectarines and peaches with yellow flesh, in contrast to the white-flesh peaches typical of the rest of China (Scorza, 1991).

The crop spread to Europe more than 2,000 years ago, along ancient trade routes through Persia (Hesse, 1975; Byrne et al., 2012). Peach was introduced to the Americas by Spanish and Portuguese settlers 400 years ago (Hesse, 1975; Scorza, Okie, 1990; Faust, Timon, 1995).

By now, peach is the second most important temperate fruit crop after apple. More than 1,000 varieties of P. persica with different phenotypic variations for various traits such as shape, fruit size, flavor, flower type, etc. have been produced worldwide. According to FAO data (https://www.fao.org/faostat/ en/#data/QCL), peach is now grown almost everywhere on all continents except Greenland, northern regions of Europe and some regions in central Africa (see the Figure).

There is little information in the literature about existing peach collections in the world. In the United States, the first breeding programs appeared in the late 18th century (Hesse, 1975; Scorza, Okie, 1990; Faust, Timon, 1995). Since the founding of the USDA (United States Department of Agriculture) in 1889, more than 2,100 clones or seeds of peaches and nectarines were imported into America from China and other parts of the world. The collection housed at the USDA Plant Introduction Garden at Chico, in California, had about 700 unique peach accessions. It was used by most breeders but focused primarily on genotypes derived from crosses with a member of the southern group of Chinese cultivars, ‘Chinese Cling’ (Scorza et al., 1985). This variety was widely used for crop improvement. During the 1950–1960s, the collection gradually declined and was almost eliminated with the closure of the station in the late 1960s. Only 60 varieties survived and were transferred to farms in Byron, Ga., and Beltsville, Md. farms. These varieties have unique traits not found in the gene pool descending from ‘Chinese Cling’ (Werner, Okie, 1998). Currently, the gene pool of the peach population in the U.S. is considered to be the most impoverished.

In order to establish a peach cultivar collection in China, peach germplasm collection was initiated at the Zhengzhou Fruit Research Institute (ZFRI) in the 1960s. In 1986, the National Peach Collection was established, which consisted of more than 600 accessions by 2000 (Wang et al., 2001). To date, more than 1,200 peach accessions have been collected from around the world, including wild species, ancient cultivar populations, and modern cultivars (Lirong et al., 2020).

Two peach collections are located in northern Spain: the National Peach Collection Gene Pool “Centro de Investigación y Tecnología Agroalimentaria de Aragón” (CITA) and “Estación Experimental de Aula Dei” (EEAD-CSIC) (https:// cita-aragon.es/en/history-mission-vision-and-aims/). The quantitative characteristics of the collections are not presented on the website

In Russia, the largest peach collection is located in the Nikita Botanical Garden in the Crimea and has 790 peaches and 85 nectarines (Smykov et al., 2021).

Peach is diploid (2n = 16), self-pollinating, with a base chromosome number of eight and belongs to the Rosaceae family, subfamily Prunoideae (Bassi, Monet, 2008). It has a lower level of genetic variability compared to other Prunus cultivars. On the one hand, the ability to self-pollinate is a limiting factor in breeding programs, on the other hand, in combination with such biological features as small genome size (265 Mb) (Arumuganathan, Earle, 1991) and diploid set of chromosomes, and as a result of its economic value, peach is an excellent model for genomic studies of stone fruits of the Rosaceae family (Monet et al., 1996; Abbott et al., 2002). The genomes of different Prunus species are highly conserved (Dirlewanger et al., 2004), allowing many of the major genes and loci of peach and other Prunus species to be placed on the same genetic map (Abbott et al., 2008).

Traditional peach seedling breeding is a labor-intensive process that takes 10–15 years from the initial crossing to the emergence of a new cultivar (Bliss, 2010; Ru et al., 2015). In addition, peach breeding programs require significant acreage due to the large size of the trees, as well as financial resources to cover the ongoing costs for technical treatments such as spraying herbicides, insecticides and fungicides, planting, pruning, thinning and watering. With the development of genetics, studies on the genetic diversity of the crop began (Herrero et al., 1964), the use of diagnostic DNA markers was developed (Callahan et al., 1991; Lambert et al., 2016; Demirel et al., 2024), and the advent of NGS sequencing techniques (Micali et al., 2015; Kim et al., 2021) allowed the generation of high-quality whole genome sequences for genomic breeding approaches.

The aim of this review is to summarize the results of genetic and breeding works for the P. persica culture based on the application of NGS methods.

## Methods before NGS: isoenzymes, DNA markers, first linkage maps

Despite the significant progress made by peach breeders over the past hundred years, traditional seedling breeding is a labor-intensive process since, in temperate climates, peach trees require at least three years to reach fruiting maturity before progeny fruit quality can be evaluated (Bliss, 2010). In the late 1980s, it was recognized that markers, the alleles of which have distinct differences at the phenotypic level, could be useful in the analysis of complex traits (Monet, 1988). And markers, which are closely linked to traits that appear late in development, can be valuable for early tree selection (Chaparro et al., 1994).

Research on peach varieties as donors of desirable traits began in the 1980s (see the Table). Protein markers, or isozymes (isoenzymes), were the first to be used as potential markers to identify particularly valuable hybrids. Isoenzymes are different variants of an enzyme (different amino acid sequence isoforms of the same enzyme) that differ in electrophoretic mobility. Isoenzyme analysis could be used to distinguish hybrids between plum and peach from plum offspring (Parfitt et al., 1985), peach and almond hybrids (Arulsekar et al., 1986а; Chaparro et al., 1987). G.E. Jr. Carter with colleagues showed that differences in protein structure were sufficient to distinguish each peach cultivar (Carter, Brock, 1980) or to reveal their similarities (Arulsekar et al., 1986b).

**Table 1.**
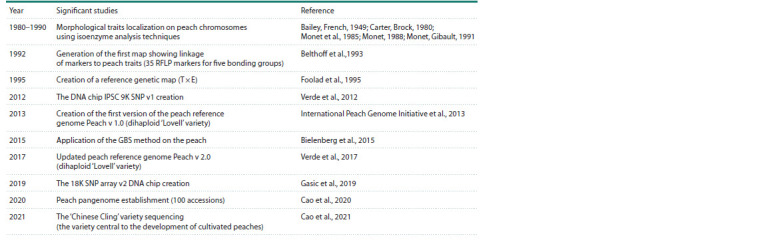
History of the genetic and genomic P. persica research

Over time, a sufficient number of morphological markers, the localization of which on the chromosome is known, have become available. In this case, by analyzing F2 populations from crosses, it is possible to determine the chromosomal positions of isozyme loci relative to morphological markers. R.E. Durham with colleagues identified the presence of separate, independently inherited loci by examining isoenzymes such as diaphorase, malate dehydrogenase and peroxidase (Durham et al., 1987). A total of four phenotypic trait linkage groups with isoenzymes were known in the early 1990s (Bailey, French, 1949; Monet et al., 1985; Monet, 1988; Monet, Gibault, 1991). However, low frequency and other drawbacks have prevented the widespread use of protein markers in breeding programs.

With the development of sequencing methods, a technological breakthrough occurred, and new methods of DNA polymorphism analysis appeared, which led to the emergence of molecular markers characterized by high frequency of occurrence in the genome. A molecular marker (DNA marker) corresponds to a gene or genomic region having different variants (alleles) and associated with different phenotypic manifestations (Khlestkina, 2014). Based on their use, approaches have emerged that complement classical breeding methods by searching for DNA markers associated with valuable traits in order to accelerate the breeding process

Economic traits such as productivity, quality, maturity, tolerance to biotic and abiotic stresses are quantitative traits and are polygenic. The search and labeling of polymorphic loci associated with quantitative traits, or, in other words, Quantitative Trait Loci (QTL), is an agronomically important task. By accumulating information on molecular markers, it is possible to create genetic maps, the purpose of which is to identify neutrally inherited markers in close proximity to genetic determinants (loci or genes) that control the manifestation of certain traits, including quantitative traits (Chesnokov, Artem’eva, 2011).

QTLs for traits such as ripening period, fruit weight, size and texture, pH, titratable acidity and soluble solids have been found in peach (Quarta et al., 2002). Fruit quality QTLs tend to cluster in several genomic regions, especially in linkage groups 4, 5 and 6 (Dirlewanger et al., 2009). Similarly, most QTL and disease resistance genes are also clustered (Pflieger et al., 1999). This observation suggests that (1) a small number of Mendelian factors can explain most of the genetic variability in fruit quality traits and (2) traits of different characteristics often share common QTLs (Dirlewanger et al., 1999). Consequently, common QTLs usually correspond to different closely related genes or to a single gene with pleiotropic effects on many traits influenced by the same physiological process (Quilot et al., 2004).

Linkage maps approximate the genomic position and genetic distances between markers using linkage analysis of genetic data (Paterson, 1996; Jones et al., 1997; Collard et al., 2005). The construction of a genetic linkage map is based on meiotic events, where genetic recombination occurs, leading to the development of recombinant genotypes. The lower the recombination frequency between molecular markers, the more likely they are to be linked and in the same linkage group (Paterson, 1996; Jones et al., 1997; Collard et al., 2005). Markers are called unlinked if their recombination frequency is greater than 50 % and, thus, they are located in different linkage groups. Recently, such calculations have been done using software, e. g. MapChart 2.2 (Voorrips, 2002), Mapmaker (Lander et al., 1987), TMAP (Cartwright et al., 2007), MapQTL (Van Ooijen, 2009), Joinmap (Van Ooijen, 2006).

The first maps showing the association between peach phenotypic traits and markers appeared in 1992 (Belthoff et al., 1992). L.E. Belthoff and colleagues (1992) developed a genetic map containing five linkage groups using 35 RFLP markers. J.X. Chaparro and colleagues developed a linkage map for peach using 83 RAPD markers and two isozymes (Chaparro et al., 1994). Then, by investigating the F2 generation obtained from crosses between almond (cultivar ‘Texas’) and peach (cultivar ‘Early Gold’), a genetic map (T × E) was created, which was later used as a Prunus reference map (Foolad et al., 1995; Joobeur et al., 1998; Pozzi, Vecchietti, 2009). It included all eight clutch groups and covered a total distance of 491 cM. Microsatellite markers (Simple Sequences Repeats, SSR) have been widely used as diagnostic DNA markers in peach research since the 2000s (Sosinski et al., 2000; Dirlewanger et al., 2002; Hong et al., 2013).

The Prunus ‘T × E’ reference map contains 1,947 anchor markers (i. e., evenly distributed throughout the Prunus genome) with known map locations (Howad et al., 2005; Dirlewanger et al., 2007; Pozzi, Vecchietti, 2009), which allowed comparisons between the peach genomes and the rest of the Prunus species. This facilitated the subsequent development of intraspecific maps for peach and other maps of interspecific relationships in Prunus (Howad et al., 2005; Dirlewanger et al., 2007; Pozzi, Vecchietti, 2009).

Currently, 70 linkage maps have been generated for peach and related interspecific hybrids, which can be found in the Rosaceae Genome Database (GDR; http://www.rosaceae.org/) (Jung et al., 2008, 2014) as well as in J.A. Salazar et al. (2013). These genetic linkage maps continue to serve as effective tools for comparison with the Prunus ‘T × E’ reference map

## Peach genomic studies

To identify and localize loci, with the following identification of candidate genes under the control of an economically valuable qualitative or quantitative peach trait, it is necessary to obtain the complete genome of P. persica (Tanksley et al., 1989; Winter, Kahl, 1995; Paterson, 1996; Jones et al., 1997; Collard et al., 2005).

The first Peach v1.0 genome variant was submitted in 2013 (International Peach Genome Initiative et al., 2013). The target for the full genome sequencing was a dihaploid genotype of the cultivar ‘Lovell’ (Plov2_2N), which was read by the Sanger method with 8.5-fold coverage. Eight pseudomolecules, reflecting the eight peach chromosomes, were assembled. The resulting genome assembly (Peach v1.0), spanning 227.3 Mb, of which 218.4 Mb (96 %) was decoded, contained 27,852 annotated genes, with an average density of 1.22 genes per 10 Kb. In 2017, an updated Peach v2.0 reference map was published (Verde et al., 2017), constructed by repeated NGS sequencing of the ‘Lovell’ dihaploid sample on the Illumina platform. The sequence totaled 227.4 Mb, which is only slightly longer than the first variant, but deeper resequencing resulted in 225.7 Mb (99.2 %) of the sequence being decoded. The approximate positions of the centromeric regions of the chromosomes were determined based on repetitive regions with low gene concentration and low recombination frequency. Based on the reference genome sequence, researchers studied the evolutionary history of the peach fruit (Cao et al., 2014, 2020; Yu Y. et al., 2018), identified domestication regions (Cao et al., 2014; Akagi et al., 2016; Li Y. et al., 2019), and genes controlling economically valuable traits (Cao et al., 2014, 2016, 2019).

The Peach v2.0 physical map was correlated with four genetic maps: (1) 67 forms from a mapping population of an interspecific cross between almond and peach ‘Texas’ × ‘Earligold’ F2 (T × E (Joobeur et al., 1998)); (2) 242 forms from a mapping population derived from IF7310828 × Ferganensis BC1 (P × F (Dettori et al., 2001)); (3) 305 seedlings from the mapping population ‘Contender’ × ‘Ambra’ F2 (C × A (Eduardo et al., 2011)); (4) 62 hybrids from the cross ‘Maria Dolce’ × SD81 F1 (MD × SD). The mapping strategy involved an approach using SSR and SNP markers targeting specific regions of the peach genome and a full-genome approach using the IPSC 9K SNP v1 chip (Verde et al., 2012). The loci derived from the genetic maps were mapped to physical positions using the MareyMap package (Siberchicot et al., 2017). For each linkage map used in this study (T × E, C × A, P × F and MD × SD), recombination rates were estimated as the ratio between genetic (cM) and physical (million base pairs) distances.

However, a single reference assembly does not reflect intraspecific variability, so there is a need to investigate the genetic variation of different peach cultivars and their wild relatives using pangenomic analysis. Similar work on pangenome construction has been carried out for many crops such as soybean (Li Y.H. et al., 2014; Liu Y. et al., 2020), rice (Zhao et al., 2018), sunflower (Hübner et al., 2019), tomato (Gao et al., 2019), barley (Jayakodi et al., 2020). In 2014, several accessions’ genomes were comparatively analyzed. To assess the process of peach domestication, 11 peach accessions (including the dihaploid ‘Lovell’ PLov2-2N used for reference assembly as a control) and one each of P. ferganensis, P. kansuensis, P. davidiana and P. mira were re-sequenced. P. ferganensis is considered a wild undomesticated peach or, more likely, represents an intermediate variant in the peach domestication. Using a set of 953,357 high-quality SNPs identified in P. persica and P. ferganensis samples, nucleotide sequence diversity was assessed for eight collected chromosomes (International Peach Genome Initiative et al., 2013).

In the first chromosome, the number of genetic variants at polymorphic loci was minimal. The greatest diversity among SNPs was observed in the distal region of the short arm of chromosome 2 and in the distal region of the long arm of chromosome 4. The density of genes encoding receptor proteins from the family of conserved nucleotide-binding leucinerich proteins (R-proteins), which are involved in immunity, was 5-fold higher on chromosome 2 than in the rest of the genome (Dodds, Rathjen, 2010). Immunity-related regions are rapidly evolving, so the diversity detected is natural. It is known from the literature that genes associated with fruit ripening are located on chromosome 4 (Eduardo et al., 2011; Dirlewanger et al., 2012).

Since the study included samples with different ripening times, there is a high level of variability in the region associated with ripening due to the given sampling parameters. However, genotypes of P. kansuensis, P. davidiana or P. mira mature at the same optimal time and no SNP diversity between regions was found in these species (International Peach Genome Initiative et al., 2013). Similarly, studies by sequencing of six related peach (P. persica) accessions were also conducted (Guan et al., 2019). The genomic variations identified showed that the comparison of different crop genotypes is effective for the development of DNA markers. These works support the need to analyze more accessions, as there are still insufficient data to identify the polymorphic regions of the genome.

The first peach pangenome, consisting of 100 sequenced samples of P. persica, was obtained in 2020 (Cao et al., 2020). Also, in this work, the de novo genomes of four wild peach relatives, P. mira, P. davidiana, P. kansuensis and P. ferganensis, were assembled. When the sequenced peach accessions were compared with the reference genome (Verde et al., 2017), an average of 3.4 % of reads in each accession failed to match the reference genome, and these reads were assembled de novo by the researchers. In total, an additional 2.52 Mb of new sequences containing 2,833 contigs (>500 bp) of potential significance were obtained. Additionally, 923 new genes were identified in the newly assembled sequences (Cao et al., 2020). The total number of genes in the pangenome was 27,796. Genes were divided into conserved genes which were common to all 100 samples (24,971, 89.9 %), and variable genes (2,803, 10.1 %), the presence of which was detected in less than 99 % of the samples (Cao et al., 2020).

Pangenomic analysis revealed the presence of resistance genes (R-genes) among the variable gene set. A similar situation has been observed in soybean (Li Y.H. et al., 2014) and rice (Zhao et al., 2018). It is hypothesized that variations in resistance (R) gene copy number may help explain differences in resistance between wild and cultivated accessions (Li Y.H. et al., 2014). Also, using peach pangenome, we found that 63 % of ornamental, 88 % of local, and 91 % of improved cultivars had a set of “optional” four genes encoding geraniol- 8-hydroxylases, which are involved in the biosynthesis of terpenes, which play an important role in plant life and have anticarcinogenic, antiseptic and antimicrobial effects. These genes may have been under positive selection pressure both during domestication and during the breeding process

When comparing P. persica with four wild species of the genus Prunus collected de novo by K. Cao and colleagues, it was found that 34.7 % of all genes found based on homology for encoded proteins were represented in all five species. At the same time, species-specific genes were found in P. mira (543 specific genes), P. davidiana (485), P. kansuensis (194), P. ferganensis (197), and P. persica (320). Such studies allow the identification of genes that confer species-specific properties. For example, a nematode resistance gene was identified in P. kansuensis (Cao et al., 2020). Such work makes it possible to identify differences in the genomes of closely rela- ted species and varieties, which is necessary for the identification of genes responsible for valuable qualities and traits of plants.

In 2021, K. Cao and colleagues (Cao et al., 2021) sequenced the ‘Chinese Cling’ cultivar, which is very important historically and central to the cultivated peach development in Europe (Byrne et al., 2000), Japan (Yamamoto et al., 2003) and the USA (Aranzana et al., 2010). The assembled genome contained 247.33 million base pairs, representing 99.8 % of the putative genome. Its comparison with the ‘Lovell’ reference genome revealed 685,407 novel SNPs, 162,655 insertions and deletions, and 16,248 copy number variation (CNV) structural variants. Gene family analysis revealed a reduction in gene families involved in the biosynthesis of flavones, flavonols, flavonoids and monoterpenoids compared to the ‘Lovell’ variety genome.

Thus, the genomic approach allows the comparative analysis of varieties and identification of variable genes (or loci in the genome) that may be responsible for different varietal traits. Such studies remain essential for further development of genomic selection in peach.

## New approaches in peach research with NGS

Polymorphism analysis methods have evolved from rather labor-intensive isoenzyme-related and RFLP methods to highthroughput sequencing methods. Comparative studies of genetic distances between peach accessions estimated using SNP and SSR markers have been conducted. In the early 2000s, methods using SSR markers became dominant. M.T. Hamblin and colleagues showed that 89 SSR markers did a better job of clustering the samples of the study sample of 259 maize inbred lines than a set of 847 SNP markers. The researchers concluded that a large number of polymorphic single nucleotide loci are needed for qualitative analysis using SNP markers (Hamblin et al., 2007). Currently, tens of thousands of SNP loci are being analyzed. J.M. Yu and colleagues (2009) calculated that the power of 1,000 SNPs is similar to that of 100 SSRs for estimating population structure and relatedness. At the same time, SSR markers remain a major option for screening plant genetic resource collections (Nybom, Lācis, 2021) and for passporting samples (Trifonova et al., 2021).

SNP markers have a higher distribution frequency in the genome compared to SSR markers, which makes them more functional when polymorphisms within specific genes are required for targeted studies. The first SNP detection technologies were in silico search for SNPs by analyzing EST databases followed by PCR-based validation (Batley et al., 2003), and SNP detection by resequencing transcripts using the Sanger method (Morozova, Marra, 2008). However, these methods were unable to detect SNPs in intergenic and non-coding regions. The advent of GBS approaches and the development of DNA chips have overcome the problems associated with the low throughput ability and high cost of SNP detection (Mardis, 2008) and now allow cost-effective and time-efficient detection of SNPs at significant loci. More and more diagnostic SNP markers are now being used in breeding programs. The use of insertions/deletions as markers is also common, but their reproducibility is lower than that of SNPs.

One common approach to SNP determination is genoty- ping using microarrays, or DNA chips. SNPs on the chip have been developed in such a way that it is possible to differentiate the samples under investigation in the pool of samples. DNA chips have been developed for many commercially important crops on two different platforms: the Illumina Infinium platform (6K for cherry (Peace et al., 2012), 8K for apple (Chagné et al., 2012), 18K for grape (Laucou et al, 2018) and 6K for avocado (Kuhn et al., 2019)) and the Axiom platform (480K SNPs for apple (Bianco et al., 2016), 68K for rose (Koning-Boucoiran et al., 2015), 700K for walnut (Marrano et al., 2019), and 70K and 200K for pear (Montanari et al., 2019; Li X. et al., 2019)).

In order to establish the medium-density Infinium SNP platform suitable for genotyping the peach gene pool, 56 breedingsignificant peach accessions spanning the crop gene pool were selected. The samples selected were those used in international peach breeding programs, contributing to the breeding gene pool according to pedigree records, and based on parentage estimates from SSR studies showing genetic diversity. Over 1 million SNPs were obtained and tested, of which exactly 9,000 passed quality control, were genetically informative and formed the platform for genotyping, the first International Peach SNP Consortium (IPSC) peach 9K SNP array v1 chip (Verde et al., 2012). SNPs on the chip were distributed evenly across all eight chromosomes and the average spacing was 26.7 bp (Verde et al., 2012).

Platform validation was performed on 709 peach accessions comprising two independent evaluation samples: 232 accessions from the European Union and 479 accessions from the USA. The EU panel included 229 peach cultivars, and three wild species of the genus Prunus or their hybrids with peach. The US panel included pedigree varieties, breeding lines, and seedlings. Overall, the sampled material consisted of 45 % cultivars, 4 % improved breeding lines, and 51 % seedlings. Specimens clearly related to either peach or almond accounted for 82 and 2 %, respectively, while 16 % of the genotyped material was of interspecific origin (with almond).

In the next step, the peach 9K SNP array v1 platform was extended to 18K. The new chip included 9,000 SNPs from the previous version and 7,206 SNPs identified by sequencing 49 samples and uniformly distributed across all peach chromosomes (Gasic et al., 2019). The uniform distribution of polymorphisms selected for the chip throughout the genome (the number of gaps smaller than 0.3 million base pairs reduced to 2 on the chromosomes 3 and 8) allows finding associations linked to the traits of interest.

Currently, genotyping by sequencing (GBS) has become the most common method of analyzing SNP markers for genome research. The term “GBS” is already used as an umbrella term for various NGS-based high-throughput genotyping methods under development (Rasheed et al., 2017). In plants, this method was first described by R.J. Elshire et al. in 2011 (2011).

Genotyping methods are used both for sequence determination and to identify associations between phenotype and genotype. Since the peach genome has now been sequenced, the identification of genomic regions associated with a trait can be performed immediately to search for candidate genes. The GBS method has been applied in peach research since 2015 (Bielenberg et al., 2015). Research in quantitative genetics is conducted equally using GBS (Cao et al., 2016, 2019; Guan et al., 2019; Li Y. et al., 2019; Meng et al., 2019; Guajardo et al., 2020; Thurow et al., 2020; Huang et al., 2021; Liu J. et al., 2021; Tan et al., 2021; Li X. et al., 2022, 2023), as well as using SNP chips (Micheletti et al., 2015; Akagi et al., 2016; Font i Forcada et al., 2019; Cirilli et al., 2021; da Silva Linge et al., 2021; Fu et al., 2021; Mas-Gómez et al., 2021, 2022).

Both GBS and SNP-chip genotyping have their advantages and disadvantages. For example, the diversity of biallelic SNPs collected at chip creation is limited, while the GBS method can cover and identify significant SNPs associated with a trait that are, however, not included in the chip set. Conversely, GBS often includes a large amount of missing data and coverage must be high enough to ensure reproducibility between the samples studied (Nybom, Lācis, 2021). GBS is currently used more frequently than SNP chips because this approach can be applied to crops for which the reference genome has not yet been sequenced. At least 96 samples are required for large-scale genotyping with GBS or with SNP chips (Zurn et al., 2020).

## Analysis of associations between genomic loci and phenotypic traits

Today, modern technologies make it possible to perform genome-wide association studies (GWAS), the results of which are effectively used in breeding programs because they allow simultaneous genomic analysis of several hundred varieties for tens of thousands of loci, comparing the associations between different alleles and the trait of interest. By creating an appropriate sample, GWAS can identify loci for several economically valuable traits at once. This step expands the ability to select markers for agronomically important traits. In the future, the use of molecular markers will allow the selection of desired genotypes among breeding hybrids, which is actively used in marker-assisted breeding (MAB) programs (Khlestkina, 2014). The identification of significant associations facilitates the development of new markers, which can be used to set the required criteria for the variety to be developed.

In peach populations, due to the low level of genetic diversity, association mapping must consider linkage disequilibrium (LD), which is the non-random relationship between two alleles that causes certain allelic combinations to occur most frequently. The method is sensitive to the presence of a large number of related samples in the population structure, leading to spurious associations between phenotypes and marker loci (Mariette et al., 2010). Thus, if a particular combination of alleles confers an adaptive advantage, its frequency will increase relative to the frequency expected under random assignment. Several studies using SSR markers have been conducted in peach in varieties with different genetic backgrounds, and their results indicate that linkage disequilibrium is quite high in this crop.

Kinship between varieties and selection increase the level of linkage disequilibrium. It has been found to range from 6.01 to 20 cM (Aranzana et al., 2010; Cao et al., 2012; Font i Forcada et al., 2013). One strategy to deal with high linkage disequilibrium is to use SNPs that are not correlated with each other for analyses (e. g., taking r2 = 0.20 as a measure of allelic association). Several algorithms exist to prune SNPs in this way or to reduce the degree of linkage disequilibrium between SNPs. Popular pruning strategies are implemented in PLINK 1.07/1.9 (Purcell et al., 2007), which sequentially scan the genome for correlated SNP pairs using only allele counting. As a result, only one representative SNP is retained for each region where highly correlated SNPs are present (Joiret et al., 2019).

The GWAS method has now identified genomic regions associated with many peach traits. Agronomic traits such as maturation, fruit pubescence, flesh colour, texture, flesh colour around the stone, fruit weight and soluble solids content are being studied (Micheletti et al., 2015; Cao et al., 2016; Elsadr, 2016; Font i Forcada et al., 2019; Li Y. et al., 2019; Liu H. et al., 2019; Thurow et al., 2020; Cirilli et al., 2021; da Silva Linge et al., 2021; Mas-Gómez et al., 2021, 2022; Tan et al., 2021; Li X. et al., 2023), as well as seed characteristics (kernel flavor) (Cao et al., 2016), pollen fertility traits (Huang et al., 2021), flower characteristics (Micheletti et al., 2015; Cao et al., 2016; Elsadr, 2016; Meng et al., 2019; Tan et al., 2021). There are works on peach resistance to various diseases (Fu et al., 2021; Li X. et al., 2022), cold and drought tolerance (Li Y. et al., 2019; Tan et al., 2021).

The above works demonstrate the potential value of the GWAS method for identifying new genomic regions associated with phenotypic traits of agricultural importance. This method can also be used to refine data on previously discovered QTLs (e. g., to more accurately determine the size of the locus under study) and facilitate the discovery of genes controlling the trait under investigation.

## Conclusion

With the development of NGS approaches, several peach cultivars have been sequenced, providing a basis for wholegenome association studies. The large diversity of cultivars in existing collections allows not only to assess the diversity of the crop’s gene pool, but also to search for marker-trait associations. Modern genotyping methods using GBS and SNP chips allow the identification of new markers that enrich the peach database. On the one hand, these new associations are of fundamental interest, contributing to the identification of peculiarities of genome evolution, individual development of the peach tree and mechanisms of response to various environmental stimuli, and on the other hand, they are the basis for applied work aimed at developing effective markers and their use in obtaining new peach varieties with specified characteristics. This approach makes it possible to accelerate the breeding time of this stone fruit.

However, difficulties remain in the field of association mapping in peach breeding programs. This is mainly due to the fact that the number of samples in the collections studied should be at least 100 to reflect the degree of efficiency. In addition, the relatedness of the varieties and hybrids under study should be assessed beforehand when compiling the sample set. Thus, when working with peach collections, preliminary analysis of genetic diversity and relatedness is necessary, which is also better performed using SNPs

## Conflict of interest

The authors declare no conflict of interest.
